# The Tenchini’s collection: a forensic anthropometric legacy of 19th century Parma, Italy

**DOI:** 10.1080/20961790.2018.1541501

**Published:** 2019-02-14

**Authors:** Laura Donato, Roberto Toni, Alessandro Porro, Marco Vitale, Fulvio Barbaro, Rossana Cecchi

**Affiliations:** a Department of Medicine and Surgery, Section of Forensic Pathology, University of Parma, Parma, Italy;; b Department of Medicine and Surgery, Museum and Historical Library of Biomedicine (BIOMED), and Section of Human Anatomy, University of Parma, Parma, Italy;; c Department of Clinical and Community Sciences, University of Milan, Milan, Italy;; d Department of Medicine and Surgery, Section of Human Anatomy, University of Parma, Parma, Italy

**Keywords:** Anatomical collection, death mask, forensic sciences, inmates, multidisciplinary, Parma

## Abstract

A group of 19th century inmates dead in the prison of Parma are the protagonist of an incredible scientific collection. Lorenzo Tenchini started the creation of this collection and dedicated his work and his studies to its completeness. Anatomist and academic, Lorenzo Tenchini (1852–1906) dedicated his scientific studies to macroscopic anatomy, particularly about central nervous system and its correlation with psychic function. In 1881 he became ordinary professor in Normal Human Anatomy at the University of Parma dedicating himself to the study of the anatomical organization of the brain and psychic and social disturbs. During the study of the skulls and brains of psychotic patients and the deformations of skulls belonging to patients admitted in the Hospital of Brescia, he started a collaboration with Alessandro Cugini (1829–1913), founder of the Institute of Legal Medicine at the University of Parma. Tenchini realized an anatomical collection, preserved today in the Museum of Biomedicine of the University of Parma. This collection represents the masterpiece of his research carried out during his academic activity and still a unicum in the western world, as there are no similar collection assembling such a multidisciplinary information. The peculiarity of this collection is due not only to the scientific interest of the anatomic samples and their full clinical documentation, but also to the methods employed in order to realize it. At the end of the 19th century, as a student of Cesare Lombroso (1835–1909), Tenchini based his work on the study of the face, the skull and brain of each dead inmate of Parma’s prison or Colorno’s mental hospital. These individuals as protagonists of Tenchini’s collection, leave a legacy identifiable as scientific heritage. Their skulls and brains, the reproduction of their faces through ceroplastic and other anatomical samples treated with other techniques, are accompanied by an autoptic and psychiatric full documentation, allowing the collection to be complete with every aspect related to the inmates studied. Through his work, a comparison between different kind of studies, such as psychiatry, psychology, neurology, legal medicine and anthropology, is suitable in scientific research to be realized. Moreover, data come from a forensic context: this allows a comparison with different methodologies employed in modern age by forensic expertise such as the comparison between modern and ancient medical diagnostic technique. This masterpiece represents Tenchini’s neuroanatomical research on behaviour and set a pioneering step in the history of biomedical science allowing further multidisciplinary studies.

## Introduction

In the modern age, biomedical achievements spur scientific research. Among new experimental approaches and methodologies, the ingenious and revolutionary studies of an Italian anatomist from the 19th century stand out as his scientific legacy.

A group of 19th century inmates who died in a prison of Parma, Italy, are the subjects of an incredible scientific collection created by Lorenzo Tenchini (1852–1906). As an anatomist and academic, Tenchini studied macroscopic anatomy, particularly that of the central nervous system and its correlation with mental function.

Lorenzo Tenchini studied medicine at the University of Pavia, Italy. In 1881, he became full professor of human anatomy at the university, dedicating himself to the investigation of skulls and brains of psychotic patients, initially from the psychiatry of the Hospital of Brescia. He also started a fruitful collaboration with Alessandro Cugini (1829–1913), founder of the Institute of Legal Medicine at the University of Parma.

In Parma, Tenchini’s studies focused on the corpses of inmates who died from illness (mainly tuberculosis) in the prison of Parma and in the nearby mental hospital of Colorno. His research followed the guidelines of Cesare Lombroso (1835–1909, his teacher at the University of Pavia), aimed at understanding any possible link between the phenotype, anthropometric parameters of the skull, and features of the criminal behaviour.

Tenchini’s anatomical collection, the masterpiece of his research, is preserved today in the Museum and Historical Library of Biomedicine (BIOMED) at the University of Parma. The collection is unique, not only because of the scientific interest of the anatomic samples and their full clinical documentation (no other collections have assembled such a wealth of multidisciplinary information), but also because of the methods employed to develop it. The skulls, brains and reproductions of the subjects’ faces through ceroplastics (wax modelling) are accompanied by autopsy and psychiatric reports [1].

Famous and important anatomical collections are also available at other institutions: the anatomical collections of the Universities of Halle [2] and Berlin in Germany [3,4], of Vienna in Austria [5,6] and of Bucharest in Romania [7] are all of forensic interest. These collections comprise mostly skulls, skeletons, anatomical wax models and tattoos, and they are currently used for teaching purposes. Tenchini’s collection, however, is unique, not only because of the scientific interest of the anatomic samples and their full clinical documentation, but also because of the methods used. As a student of Cesare Lombroso and a follower of his ideas, Tenchini’s research endorsed the physiognomy principles of the late 19th century; the faces, skulls, and brains of the patients and inmates he studied still provide a wealth of information from his scientific investigation. Remarkably, all his anatomical preparations (skulls, brains, mummified viscera and masks reproducing the faces of the subjects studied), are accompanied by autopsy and sometimes psychiatric documentation. They provide a composite picture of the physical and social anthropology of the subject analyzed [1].

The Tenchini’s collection includes: (1) *Antropologia Criminale* (*Criminal Anthropology*, AC) cards, (2) dried brains, (3) masks, (4) skulls and (5) dried organs.

The 131 AC cards describe the criminal events and the most relevant autopsy features of the inmates studied, including macroscopic aspects of the brain, such as weight and morphometric index. The matching of AC card with the related mask was possible in only 51 cases. The AC cards are archived in the Historical Library of BIOMED.

There are 33 dried brains that were prepared using the method of Giacomini (1840–1898) [8]. Of these, only eight could be associated with their masks. There are 14 brains that are associated with an AC cards, although they lack corresponding masks. Thus, a total of 22 brains are matched to the inmates studied by Tenchini. It is possible that the other 11 brains belong to subjects other than inmates [1]. According to the original AC cards, however, Tenchini prepared at least 131 brains collected in 7 years (between 7 June 1882 and 16 July 1889). In the years that followed, Tenchini continued to preserve brains through drying, classified in the E series, not necessarily obtained from inmates. Based on his collection and study of brains from inmates, Tenchini published his main scientific work, entitled *Cervelli di Delinquenti (Criminal Brains)*, in which he provided extensive descriptions of the main changes he had observed in the macroscopic morphology of each brain.

Of 76 masks, 46 are still viewable at BIOMED in Parma, and 30 are kept at Cesare Lombroso's Museum of Criminal Anthropology in Turin. However, all masks were originally prepared in Parma, and some of them were either donated by Tenchini himself to Cesare Lombroso, or were forgotten in Turin after Tenchini’s premature death in 1906, remaining there forever. In particular, between 27 March 1885 and 16 July 1889, Tenchini assembled an archive of the H series in Parma, in which he described the masks of 102 dead inmates. Based on the descriptive scroll on some masks, or on the back of their wooden support, Tenchini might have studied at least 156 masks [1], more than a half of which are presently missing.

Tenchini studied more than 400 skulls, including those of the dead inmates from the prison in Parma and the mental hospital in Colorno.

Tenchini collected more than 350 dried viscero-appendicular samples, mostly from the bodies of the subjects portrayed in the masks [8].

Tenchini used Giacomini’s method to preserve the brains as follows [9]: The brain is immersed in a zinc chloride solution for 4–5 days, and then in alcohol for at least 10 days. The hardened brain is transferred into glycerine, which gradually replaces the alcohol. The brain is extracted from the liquid and, once dry, a thin layer of elastic clay is spread on the surface. The brain is then rinsed in a renewed solution of 2% chromium, then in a 3%–4% solution for at least 1 month. The brain is immersed in water for a few days to remove the excess dichromate, then for 6–8 days in alcohol, and then in glycerine. The brain is hardened in 10%–12% nitric acid for 12–15 days, followed by treatment with potassium dichromate.

Professor Tenchini used a unique technique to prepare the masks (1885–1889), the details of which have partly emerged only recently. Studies are still in progress to clarify the specific steps of his procedure, which was never reported in any of his scientific papers [1]. In particular, it is now clear that a basal layer is constituted by an inorganic component, such as plaster. This layer is covered by an intermediate radiopaque material, with features similar to those of the organic tissue, including human skin and natural fibers, such as cotton, variably soaked in wax. This represents the most superficial layer of the mask, giving the final semblance to the reconstructed face.

**Figure 1.**
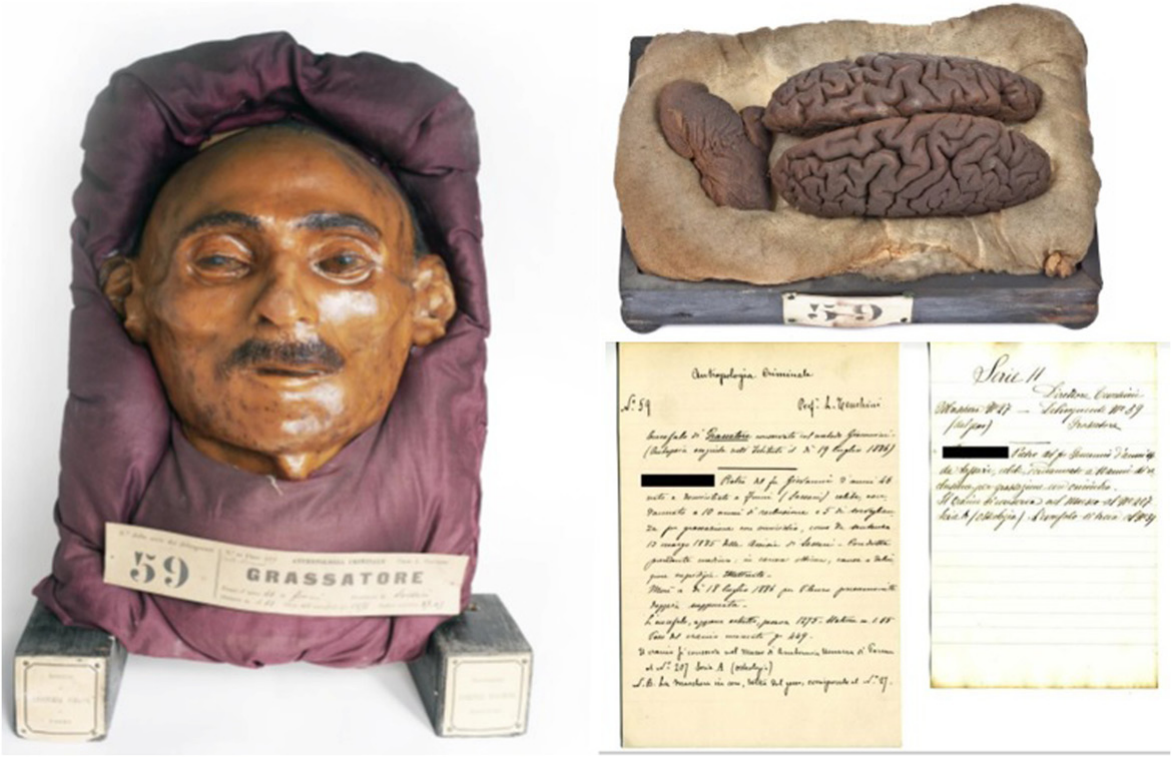
Literal translation from Italian transcription of the 19th century: Criminal Anthropology. No. 59 Prof. L. Tenchini. Robber brain conserved with Giacomini’s method. (Autopsy performed in the Institute on 19 July 1886.) P.M.P. of the late Giovanni, born 46 years old and domiciled in Fonni (Sassari), unmarried, sentenced to 10 years of imprisonment and 5 years of surveillance for fatigue with murder, as per sentence 13 March 1885 of the Assisie of Sassari. Previous conduct mediocre: excellent in prison, cause of a crime greed. Illiterate. He died on 18 July 1886 for pleuro suppurated double pneumonia. The brain, just extracted weighed 1.275 g. Stature 1.65 m. Weight of the macerated skull is 449 g. The skull is preserved in the Museum of Human Anatomy of Parma at the No. 207 series A (osteology). N.B. The wax mask, removed from the plaster, corresponds to the No. 27. (All material kept at Museum and Historical Library of Biomedicine (BIOMED) in Parma. ©2016 University of Parma).

**Figure 2.**
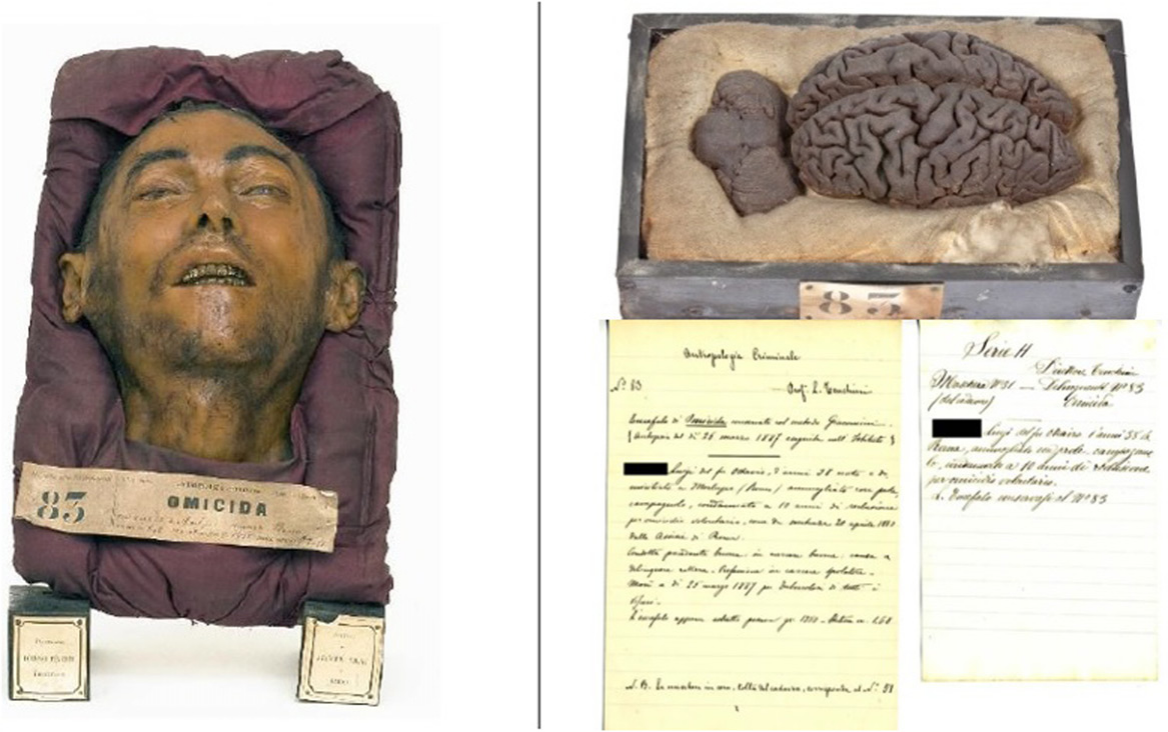
Literal translation from Italian transcription of the 19th century: Criminal Anthropology. No. 83 Prof. L. Tenchini. Homicidal brain preserved with Giacomini’s method. (Autopsy of the day 26 March 1887 performed in the Institute) M.L, 38 years old, born and domiciled in Morlupo (Rome), married with offspring, country, sentenced to 10 years in prison for voluntary homicide, as per sentence 20 April 1883 from the Assisie of Rome. Good previous conduct: good in prison, criminal cause anger. Profession in spooling prison. He died on 25 March 1887 for tuberculosis of all viscera. The freshly drawn brain weighed 1.350 g. Stature 1.68 m. N.B. The mask in wax, removed from the corpse, corresponds to the No. 51. (All material kept at Museum and Historical Library of Biomedicine (BIOMED) in Parma. ©2016 University of Parma).

**Figure 3.**
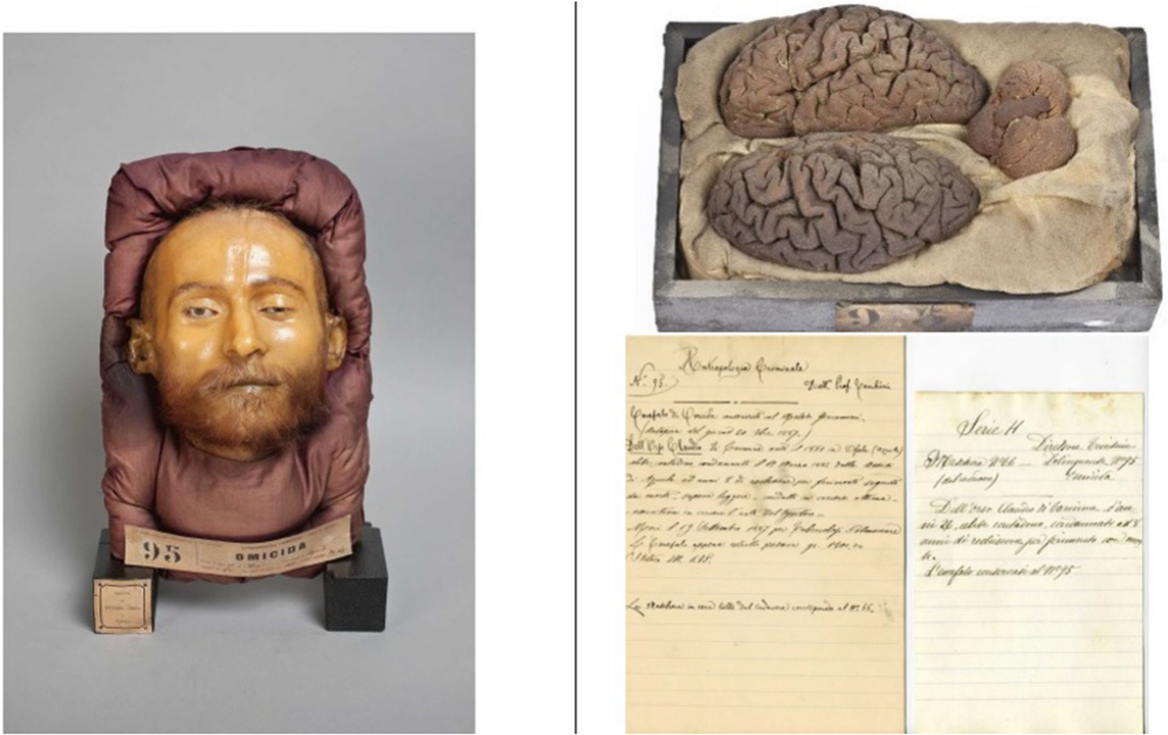
Literal translation from Italian transcription of the 19th century: Criminal Anthropology. No. 95 Prof. L. Tenchini. Brain of murderer conserved with Giacomini’s method. (Autopsy of the day “unreadable” 1887) D. C. of Carmine, born 1861 in Ofela [rectius Ofena] (Aquila), unmarried, farmer, sentenced 17 March 1885 from the Assisie of Aquila to 8 years of imprisonment for wounding followed by death – he could read – behaviour in prison excellent – he practiced the art of the weaver in prison. He died on 19 September 1887 for pulmonary tuberculosis. The brain, just extracted, weighed 1.300 g. Stature 1.75 m. The wax mask, removed from the corpse, corresponds to the No. 66. (Mask currently kept at the Cesare Lombroso’s Museum in Turin. ©2016 University of Turin).

Using microbiopsies carried out on the intermediate layer between the plaster and the wax, it has been concluded that the intermediate layer is likely organized as a sandwich, in which some cotton tissue embalmed in wax covers the plaster and, on top of this waxed cotton, the original epidermis of the cadaver has been deposited, in a sort of real “face transplant,” that is ultimately protected with a superficial layer of thickened, coloured wax. Hairs, variably inserted as a decoration into the superficial waxy layer and even partly stemming from the epidermis underneath as hair bulbs, provide an empathic feature to the reconstructed face. However, not all masks might have this structure, indeed, in some prepared between 1885 and 1886, it is not possible to detect the epidermal layer between plaster and wax (Figures 1, 2 and 3).

Tenchini also prepared many visceral and appendicular samples from the corpses. He used a drying technique similar to mummification combined with intravascular injection of coloured masses (plaster and/or jellies with vegetable gum, essential oils and mordants) [1].

## Discussion

Tenchini’s anatomical collection is a remarkable historical-scientific achievement, representing a multidisciplinary approach to the controversial hypotheses based on the biology of psychopathological and criminal behaviours.

Part of the scientific interest is focused on the particular attention that Tenchini gave to the description of the mental, social and phenomenological contexts of the inmates. Indeed, he recorded the criminal behaviour, the story, and the eventual environmental influence—including the behaviour—of their families. All these data are accompanied by the autopsy report.

One of the mysteries still under investigation is why some of the masks (and possibly also some of the dried brains) were transferred out of Parma. Indeed, 30 of them are now in Turin [10,11]. There is no evidence that these masks were prepared for educational purposes; in contrast, extensive documentation points to this aim for many of the anatomical preparations. Likely, the masks were to be connected to the physiognomy principles pursued by Lombroso, and to which Tenchini lend credence during his career. This idea pushed him to reproduce the inmates’ faces with as much realism as possible, and to use a “personalized” ceroplastic method. The dedication to this purpose is underlined by the care in maintaining the expressiveness of the faces, including their original epidermis and piliferous components, as we may read in a letter very likely addressed to Cesare Lombroso in 1906 [12–14].

The scientific value of Tenchini’s collection is related not only to its connection with Lombroso's theories, but also to current interest in the correlated masks and skulls allowing facial reconstruction. In fact, advanced studies are underway at the Institute of Legal Medicine of the University of Parma to identify an association between the masks and skulls contained in the collection, based on a facial reconstruction technique through 3D computer graphics [15]. These studies bear substantial relevance to attempts to solve another mystery of this collection: the differences in the encoding that Tenchini applied on the masks, on the dead inmates, and on the skulls. In particular, the numbers assigned to the inmates and reported on the AC cards correspond to the numbers of the descriptive scrolls applied on the masks, but they do not correspond to the numbers assigned to the masks on the archive cards. In addition, no evidence of matching is clear for the skull code. However, some correspondence has been found between the information written on the scroll and those on the skulls, such as the age at death. Therefore, we believe that the facial reconstruction will eventually allow for assignment of a skull to a mask for those that are as yet unmatched.

## Conclusion

A group of 19th century inmates who died in the prison of Parma are the protagonists of Lorenzo Tenchini’s incredible scientific collection. An impressive amount of information about each individual is recorded and organized in this collection. A description of their lives, including the social environment and the eventual psychiatric and criminal behaviour, is available. Both the life and death of those inmates were described. The circumstances, manner, and cause of death are accessible in autopsy reports, and reports on the anatomy of the inmates’ brains complete the archive.

Through Tenchini’s neuroanatomical research, multidisciplinary comparisons in psychiatry, psychology, neurology, legal medicine and anthropology are realized. Moreover, data from the forensic context allow for a comparison with different forensic methods currently employed [16–19].
